# 

**Published:** 1917-06-30

**Authors:** 


### CONTENTS.

PAGE

**Neurasthenia and the Army** ... ... ... 243

**A London Holiday for Hospital Men** ... ... 244

**Hospital and Institutional News** ... ... ... 245

The Mesopotamia Report—The System at Fault—Vice-Chancellor Sir Cooper Perry, M.D.—The United States Red Cross—Hospital Improvements at the Front—Leicester Farmers' and Graziers' Spontaneous Generosity—An Earl Kitchener Memorial—A Huge War Charity Wound Up—A New Hospital Journal—The Saturday Fund in Birmingham—A Woman Medical Officer of Health—Dentistry for Sailors and Soldiers—The Pay of Territorial Medical Officers—Founder's Day at Alton Cripples' Hospital—A Floating Hospital—The East Preston Guardians and their Infirmary—A Cottage Hospital Plan of Campaign—The Scarcity of Doctors—The Hospital Treatment of Venereal Diseases—Education and Public Health—The Voting System in Abeyance—Infant Welfare in Liverpool—What the Nurse has Won for Women—Charwomen's Pin-Money—A Prosperous Year—The End of a Series—This Week's Drug Market—To Our Readers.

**The Attacks on Medical Recruiting Officers** 251

Parliament and the Doctors.

**The Crusade Against Venereal Disease** ... 252

Addresses by Surg.-General Sir Alfred Keogh and Mrs. Creighton.

**St. Thomas's Hospital** ... ... ... ... 253

The Hon. Arthur Stanley, C.B., M.V.O., M.P., Appointed Treasurer.

PAGE

**Hospital and Institutional Results** ... ... 254

**The New Offensive Against Disease** ... ... 255

King Edward VII.'s Hospital, Cardiff.

**Parliament and the Profession** ... ... ... 255

**The Editor's Letter-Box** ... ... ... ... 256

The Chapel of Guy's Hospital—The Royal Infirmary and our Prisoners in Germany: An Example which might be Followed—Summer Knitting.

**The Matrons' and Sisters' Department** ... 257

Poor-Law Nursing in England. III. The Sick Wards.

Round the Hospitals.

Queen Alexandra's Personal Service—First Annual Meeting of the College—The Central Committee in Error—Miss Matheson Speaks Out—Trades Unions and Nursing Service not Analogous—Strawberry Teas for Hospital Nurses.

**The Past and the Outlook in Midwifery** ... 260

Report on the Physical Welfare of Women and Children.

**When the Wounded Arrive** ... ... ... 261

**Matrons' and Sisters' Appointments** ... ... iv

**Gifts and Bequests** ... ... ... ... ... iv

**The Institutional Worker** ... ... (Suppl.) 1

Electro-Medical Apparatus. Some Methods of Applying Electricity.

Question for June.

Enquiries and Answers.

